# Sociodemographic Trends and Perinatal Outcomes in Fathers 50 Years and Older

**DOI:** 10.1001/jamanetworkopen.2024.25269

**Published:** 2024-08-01

**Authors:** Albert S. Ha, Michael Scott, Chiyuan Amy Zhang, Shufeng Li, Ashkan P Langroudi, Frank Glover, Satvir Basran, Francesco Del Giudice, Gary M. Shaw, Michael L. Eisenberg

**Affiliations:** 1 Department of Urology, School of Medicine, Stanford University, Stanford, California; 2 Gangarosa Department of Environmental Health, Rollins School of Public Health, Emory University, Atlanta, Georgia; 3 Department of Maternal Infant and Urological Sciences, “Sapienza” University of Rome, Rome, Italy; 4 Department of Pediatrics, Stanford University, Stanford, California

## Abstract

**Importance:**

The age of fathers at childbirth is rising, with an increasing number of births attributed to older fathers. While the impact of advanced paternal age has been documented, sociodemographic data about fathers aged 50 years and older remain scarce.

**Objectives:**

To explore sociodemographic and temporal trends among the oldest US fathers (age ≥50 years) and their associations with perinatal outcomes.

**Design, Setting, and Participants:**

This retrospective cross-sectional study included data from all US births from 2011 to 2022 using the National Vital Statistics System. Data were analyzed from August 2023 and May 2024.

**Exposures:**

Reported paternal age at childbirth.

**Main Outcomes and Measures:**

Outcomes of interest were sociodemographic factors, temporal trends in older fatherhood, and perinatal outcomes, including preterm birth, low birth weight, gestational diabetes, gestational hypertension, assisted reproductive technology (ART), rates of maternal primiparity, and the infant sex ratio.

**Results:**

From 2011 to 2022, the US recorded 46 195 453 births, with an overall mean (SD) paternal age of 31.5 (6.8) years and 484 507 (1.1%) involving fathers aged 50 years or older, 47 785 (0.1%) aged 60 years or older, and 3777 (0.008%) aged 70 years or older. Births to fathers aged 50 years or older increased from 1.1% in 2011 to 1.3% in 2022 (*P* for trend < .001). Fathers aged 50 years or older were more diverse, with variations in educational achievement and race and ethnicity. Marital status and maternal racial and ethnic and educational backgrounds also varied by paternal age and race. Despite controlling for maternal age and other sociodemographic and perinatal factors, every 10-year increase in paternal age was consistently associated with greater use of ART (eg, age 50-59 years: adjusted odds ratio [aOR], 2.23; 95% CI, 2.19-2.27), higher likelihood of first maternal birth (eg, age 50-59 years: aOR, 1.16; 95% CI, 1.15-1.17), and increased risks of preterm birth (eg, age 50-59 years: aOR, 1.16; 95% CI, 1.15-1.18) and low birth weight (eg, age 50-59 years: aOR, 1.14; 95% CI, 1.13-1.15) compared with fathers aged 30 to 39 years. No significant changes in the infant sex ratio were observed, except among fathers aged 70 years or older (aOR, 0.92; 95% CI, 0.86-0.99) and 75 years or older (aOR, 0.84; 95% CI, 0.73-0.97), who showed a decreased likelihood of having male offspring.

**Conclusions and Relevance:**

In this cross-sectional study of all US births from 2011 to 2022, the percentage attributed to older fathers, while small, increased. Notable variations in paternal and maternal race and education were identified. Older fatherhood was associated with increased ART use, first-time maternal births, adverse perinatal outcomes, and altered sex ratio. Further research of this population is crucial for improving patient counseling and family planning.

## Key Points

**Question:**

What are the sociodemographic characteristics, temporal trends, and perinatal outcomes associated with the oldest fathers (age ≥50 years) in the US?

**Findings:**

In this cross-sectional study of all 46 195 453 live US births from 2011 to 2022, the proportion attributed to the oldest fathers increased. Compared with younger fathers, the oldest fathers were more racially and educationally diverse and associated with higher rates of maternal primiparity, use of assisted reproductive technology, and adverse perinatal outcomes.

**Meaning:**

These findings suggest that the oldest US fathers are diverse and associated with adverse perinatal outcomes; these shifts in delayed fatherhood underscore the importance of public education for family planning and counseling.

## Teaser

This cross-sectional study explores sociodemographic and temporal trends among the oldest US fathers (age ≥50 years) and their associations to with perinatal outcomes among all US live births.

## Introduction

Recent socioeconomic and demographic trends have shifted the timeline for family building in the US, with many couples increasingly delaying parenthood. Factors contributing to this delay include increased educational opportunities, career advancement, availability of contraception, and assisted reproductive technology (ART).^1,2,3^ From the maternal standpoint, advanced age has been associated with increased risk of adverse outcomes, including infertility, use of ART, low birth weight, pregnancy loss, and premature birth.^4,5^ However, technological advancements, such as oocyte donation, have enabled pregnancy potentially into the sixth decade of life.^6^

Paternal age has also affected fertility, pregnancy trajectory, and child health.^7,8,9,10^ Age-related conditions, such as erectile dysfunction and hypogonadism, impair paternal fecundity, while older age is associated with decreased semen volume, motility, and morphology.^11,12,13,14^ Recent findings also suggest age-related effects on sperm quality, including increased DNA fragmentation, sperm aneuploidy, de novo alterations, and epigenetic alterations.^11,15,16,17,18,19^ Overall, the accumulation of alterations in older men may increase the risk of conditions like autism, pediatric cancers, achondroplasia, and schizophrenia; decrease likelihood of ART success; and heighten risk of perinatal complications.^20,21,22,23,24,25,26^ Based on this evidence, the American Urological Association and American Society of Reproductive Medicine have recommended counseling couples with a male partner aged 40 years or older about the potential adverse outcomes for their offspring.^27^

While prior research has explored the clinical and biological consequences of advanced paternal age (APA), detailed sociodemographic data on the oldest fathers (eg, age ≥50 years) remain scarce. Recent Scandinavian studies have revealed socioeconomic diversity among these fathers, influenced by factors such as education and urbanization.^28,29,30^ Despite ample media coverage about older fatherhood,^31,32^ very little information about this population is known. This study aims to characterize the oldest fathers in the United States by exploring key sociodemographic trends, temporal patterns, and associations with perinatal outcomes and the infant sex ratio.

## Methods

This cross-sectional study was exempted from ethics review and informed consent by the Stanford University School of Medicine institutional review board because of the use of a publicly available, deidentified dataset. This study adhered to the Strengthening the Reporting of Observational Studies in Epidemiology (STROBE) reporting guideline for cross-sectional studies.

### Study Population

The National Vital Statistics System, overseen by the National Center for Health Statistics and Centers for Disease Control and Prevention, is a public data repository that aggregates all US birth data from standardized birth certificates.^33^ It provides detailed sociodemographic and perinatal data on families, collected from birth certificates in adherence to federal law. Sociodemographic information is self-reported by the mother, with standardized forms and procedures ensuring uniform collection.^33^

### Study Cohort and Covariates

We collected pertinent sociodemographic and perinatal data and outcomes on mothers and fathers from 2011 to 2022. Maternal data included age, race, Hispanic ethnicity, education, marital status, body mass index, insurance status, and medical history (diabetes, hypertension, prior preterm births, and smoking status during pregnancy). Key perinatal metrics include infant sex, gestational hypertension, gestational diabetes, and type of delivery. All available sociodemographic paternal covariates, including age, race, Hispanic ethnicity, and education, were analyzed. Recoded variables were used when available to minimize misclassification bias. Due to changes in racial classification starting in 2014, parental race was classified into Asian or Pacific Islander, Black, White, other (eg, American Indian or Alaska Native or multiracial) and missing to ensure consistency. Hispanic ethnicity and parental education were also reclassified (eTables 1-3 in Supplement 1). The oldest fathers with APA were defined as aged 50 years and older.

Perinatal outcomes assessed include gestational diabetes, gestational hypertension, low birth weight (defined as <2500 g), and preterm birth (defined as <37 gestational weeks). Additional outcomes include ART use, first live maternal birth, and infant sex ratio. ART encompassed any assisted reproductive technique, including pharmacologic agents and procedures, such as in vitro fertilization. Maternal primiparity was defined as the birth of the mother’s first live child, based on the maternal data on order of live births. Infant sex ratio was defined as the ratio of male to female births.

### Statistical Analysis

Paternal age was treated as both a continuous and categorical variable. Paternal age was evaluated based on the mean and SD for each birth year and dichotomized according to APA. Paternal ages were additionally segmented into 5-year (<50, 50-54, 55-59, 60-64, 65-69, 70-74, 75-79, and ≥80 years) and 10-year (<30, 30-39, 40-49, 50-59, 60-69, and ≥70 years) intervals to highlight additional trends.

Sociodemographic characteristics in older fathers were also assessed by analyzing parental age and race in relation to maternal race, parental education, and marital status (eTable 4 in Supplement 1). Stratified analyses based on paternal race were also graphically represented to illustrate sociodemographic trends. Temporal trends in APA were graphically depicted and visually evaluated by birth year. To identify a temporal association of older fathers across birth years, paternal age 50 years and older was assessed using the Jonckheere-Terpstra trend test.

Covariates such as maternal age, maternal race, parental education, insurance and marital status, maternal body mass index, and smoking status during pregnancy were selected a priori for inclusion in multivariable regression models for outcomes of low birth weight, preterm birth, first live birth, use of ART, gestational diabetes, and gestational hypertension. ART and prior preterm birth were additionally included in the models for preterm birth and low birth weight. Due to documented interactions during pregnancy,^34^ gestational and prepregnancy hypertension were incorporated into the multivariable model for gestational diabetes, while gestational and prepregnancy diabetes were included into the model for gestational hypertension. Multicollinearity was assessed by calculating the squared value of the generalized variance inflation factor (GVIF)^1/(2 × ^*^df^*^)^, setting a predetermined threshold of less than 3.^35^ Due to collinearity observed between paternal and maternal race, paternal race was subsequently omitted from the analyses.

Further subgroup analyses were performed on maternal age, given the interaction of paternal and maternal age with fertility status. Maternal age was categorized into younger than 25, 25 to 34, and 35 years or older age groups, with perinatal outcomes analyzed for each. Maternal age was also incorporated as a continuous variable in each multivariable model to control for potential confounding.

We also investigated the association of APA with the infant sex ratio. Descriptive analysis using χ^2^ tests compared paternal age younger than 50 vs 50 years or older. Sensitivity analyses further segmented paternal age into 5-year increments (≥55, ≥60, ≥65, ≥70, and ≥75 years). Logistic regression models for each paternal age group were adjusted for maternal age, maternal race, parental education, insurance and marital status, ART, gestational age, prior preterm birth, and low birth weight.

Additional investigation revealed missing paternal age data for 6 983 104 births (15.1%). The Little missing completely at random test suggested that the data were not missing completely at random. Consequently, a logistic regression model incorporating maternal age, race, and education was used to calculate inverse probability weights.^7,21,36^ These weights estimated the probability of paternal age reported for each birth and were applied in all regression analyses to enhance generalizability.^21,36,37^ Data on missing paternal age were also tabulated and presented. All statistical tests were 2-sided, with a *P* < .05 considered statistically significant. Statistical analysis was performed using R software version 4.3.1 (R Project for Statistical Computing). Data were analyzed from August 2023 to May 2024.

## Results

### Characteristics of US Fathers

From 2011 to 2022, the US recorded 46 195 453 live births, with paternal age reported in 84.9% of births (Table 1). By race, there were 2 609 655 Asian or Pacific Islander fathers (5.6%), 5 609 342 Black fathers (12.1%), 28 267 031 White fathers (61.2%), and 1 020 564 fathers (2.2%) identified as other race; 8 386 305 fathers (81.1%) identified as Hispanic (Table 1). Most fathers had some college experience (26 485 853 fathers [57.4%]) (Table 1). Additional paternal demographics stratified by race can be found in eTable 4 in Supplement 1.

**Table 1.** Baseline Demographics of US Fathers and Mothers, 2011-2022

Characteristic	Births by paternal age, No. (%)									
	All	Missing	<50 y	50-54 y	55-59 y	60-64 y	65-69 y	70-74 y	75-79 y	≥80
Total live births, No.	46 195 453	6 983 104	38 727 842	329 554	107 168	34 000	10 008	2820	692	265
**Paternal characteristics**										
Education										
<High school	5 245 299 (11.4)	20 476 (0.3)	5 151 494 (13.3)	48 747 (14.8)	16 762 (15.6)	5487 (16.1)	1622 (16.2)	515 (18.3)	133 (19.2)	63 (23.7)
High school graduate, some college, or AA	21 240 554 (46.0)	45 088 (0.6)	20 968 012 (54.1)	156 437 (47.5)	50 283 (46.9)	15 172 (44.6)	4147 (41.5)	1062 (37.7)	255 (36.9)	98 (37.0)
Bachelor’s degree	7 478 386 (16.2)	4427 (0.1)	7 388 604 (19.1)	59 926 (18.2)	17 846 (16.7)	5497 (16.2)	1562 (15.6)	395 (14.0)	106 (15.3)	23 (8.7)
≥Master’s degree	4 213 733 (9.0)	2421 (0.0)	4 137 689 (10.7)	48 754 (14.8)	16 382 (15.3)	5720 (16.8)	1966 (19.6)	625 (22.1)	132 (19.1)	44 (16.6)
Missing	8 017 481 (17.4)	6 910 692 (99.0)	1 082 043 (2.8)	15 690 (4.8)	5895 (5.5)	2124 (6.3)	711 (7.1)	223 (7.9)	66 (9.5)	37 (14.0)
Race										
Asian or Pacific Islander	2 601 655 (5.6)	106 487 (1.5)	2 452 548 (6.3)	29 051 (8.8)	9322 (8.7)	3071 (9.0)	853 (8.5)	241 (8.5)	51 (7.4)	31 (11.7)
Black	5 609 342 (12.1)	223 315 (3.2)	5 270 273 (13.6)	75 129 (22.8)	27 812 (26.0)	9169 (27.0)	2676 (26.7)	722 (25.6)	176 (25.4)	70 (26.4)
White	28 267 031 (61.2)	987 135 (14.1)	27 007 376 (69.7)	189 174 (57.4)	57 998 (54.1)	17 929 (52.7)	5342 (53.4)	1553 (55.1)	392 (56.7)	132 (49.8)
Other^a^	1 020 564 (2.2)	23 735 (0.4)	987 022 (2.6)	6686 (2.0)	2175 (2.0)	686 (2.0)	206 (2.1)	44 (1.6)	8 (1.1)	2 (0.8)
Missing	8 696 861 (18.9)	5 642 432 (80.8)	3 010 623 (7.8)	29 514 (9.0)	9861 (9.2)	3145 (9.3)	931 (9.3)	260 (9.2)	65 (9.4)	30 (11.3)
Hispanic ethnicity										
No	30 559 526 (66.2)	1 241 540 (17.8)	28 946 147 (74.7)	251 398 (76.3)	82 680 (77.2)	26 731 (78.7)	7941 (79.3)	2300 (81.6)	569 (82.2)	220 (83.0)
Yes	9 483 917 (20.5)	322 514 (4.6)	9 064 402 (23.4)	68 237 (20.7)	20 649 (19.2)	5995 (17.6)	1639 (16.4)	375 (13.3)	85 (12.3)	21 (7.9)
Missing	6 152 010 (13.3)	5 419 050 (77.6)	717 293 (1.9)	9919 (3.0)	3839 (3.6)	1274 (3.7)	428 (4.3)	145 (5.1)	38 (5.5)	24 (9.1)
**Maternal characteristics**										
Maternal age, y										
<20	4 138 019 (8.9)	1 338 648 (19.2)	2 796 638 (7.2)	1741 (0.5)	651 (0.6)	214 (0.6)	84 (0.8)	26 (0.9)	6 (0.9)	11 (4.2)
21-30	24 008 938 (52.0)	3 859 764 (55.3)	20 059 912 (51.8)	58 753 (17.8)	20 743 (19.3)	6866 (20.2)	2092 (20.9)	608 (21.6)	143 (20.7)	57 (21.5)
31-40	17 090 300 (37.0)	1 670 409 (23.9)	15 128 403 (39.1)	19 9704 (60.6)	63 707 (59.5)	20 118 (59.2)	5831 (58.3)	1611 (57.1)	389 (56.2)	128 (48.3)
41-50	958 196 (2.1)	114 283 (1.6)	742 889 (1.9)	69 356 (21.1)	22 067 (20.6)	6802 (20.0)	2001 (20.0)	575 (20.4)	154 (22.3)	69 (26)
Race										
Asian or Pacific Islander	2 916 971 (7.4)	230 562 (3.3)	2 846 283 (7.3)	44 153 (13.4)	17 091 (16.0)	6446 (19.0)	2099 (21.0)	669 (23.7)	166 (24.0)	64 (24.2)
Black	5 026 420 (12.8)	2 267 823 (32.5)	4 915 688 (12.7)	71 121 (21.6)	26 717 (24.9)	9048 (26.6)	2784 (27.8)	775 (27.5)	198 (28.6)	89 (33.6)
White	30 181 988 (77.0)	4 075 362 (58.3)	29 890 449 (77.2)	206 511 (62.7)	60 743 (56.7)	17 653 (51.9)	4892 (48.9)	1314 (46.6)	317 (45.8)	109 (41.1)
Other or missing^b^	1 086 970 (2.8)	409 357 (5.9)	1 075 422 (2.8)	7769 (2.3)	2617 (2.4)	853 (2.5)	233 (2.3)	62 (2.2)	11 (1.6)	3 (1.1)
Hispanic ethnicity										
No	34 873 484 (75.4)	5 329 571 (76.3)	29 173 409 (75.4)	250 618 (76.0)	82 332 (76.8)	26 638 (78.3)	7885 (78.8)	2271 (80.5)	548 (79.2)	212 (80.0)
Yes	10 927 474 (23.7)	1 592 378 (22.8)	9 229 550 (23.8)	73 526 (22.4)	22 806 (21.3)	6683 (19.7)	1890 (18.9)	476 (16.9)	124 (17.9)	41 (15.5)
Missing	394 495 (0.9)	61 155 (0.9)	324 883 (0.8)	541 (1.6)	2030 (1.9)	67 (2.0)	233 (2.3)	73 (2.6)	20 (2.9)	12 (4.5)
Education										
<High school	6 098 844 (13.2)	1 473 631 (21.1)	4 546 342 (11.7)	51 546 (15.6)	18 401 (17.2)	6212 (18.3)	1909 (19.1)	590 (20.9)	147 (21.2)	66 (24.9)
High school graduate, some college, or AA	23 764 953 (51.5)	3 495 120 (50.1)	20 044 489 (51.8)	151 745 (46.1)	51 013 (47.6)	16 123 (47.4)	4716 (47.1)	1316 (46.7)	321 (46.4)	110 (41.5)
Bachelor’s degree	8 889 213 (19.2)	218 930 (3.1)	8 571 022 (22.1)	69 896 (21.2)	20 584 (19.2)	6366 (18.7)	1797 (18.0)	474 (16.8)	101 (14.6)	43 (16.2)
≥Master’s	5 230 993 (11.3)	77 021 (1.1)	5 086 398 (13.1)	47 654 (14.5)	14 011 (13.1)	4208 (12.4)	1253 (12.5)	327 (11.6)	88 (12.7)	33 (12.5)
Unknown or missing	2 211 450 (4.8)	1 718 402 (24.6)	479 591 (1.3)	8713 (2.6)	3159 (2.9)	1091 (3.2)	333 (3.3)	113 (4.0)	35 (5.1)	13 (4.9)

Abbreviation: AA, Associate’s degree.

^a^ Other paternal race includes American Indian, Alaskan Native, and multiracial.

^b^ Other or missing maternal race includes American Indian, Alaskan Native, multiracial, or unknown or not stated.

Of all US births, 38 727 842 births (83.8%) births were fathered by men younger than 50 years, while 484 507 births (1.1%) were to fathers aged 50 years or older. The mean (SD) age of all fathers was 31.5 (6.8) years and 53.8 (4.2) years for those aged 50 years or older. From 2011, the mean (SD) age of all fathers was 30.8 (6.9) years, with APA fathers having a mean (SD) age of 53.6 (4.0) years. By 2022, the mean (SD) ages had increased to 32.1 (6.7) years for all fathers and 54.0 (4.3) years for APA fathers.

When stratified by race, the mean (SD) ages were 34.4 (6.0) years for Asian or Pacific Islander fathers (53.8 [4.2] years for those aged ≥50 years); 31.0 (7.8) years for Black fathers (54.1 [4.3] years for those aged ≥50 years), and 31.5 (6.6) years for White fathers (53.7 [4.1] years for those aged ≥50 years). Overall, the number of births fathered by APA fathers diminished with each 5-year increment in age, from 10 008 births (0.02%) for fathers aged 65 to 69 years to 2820 births (0.006%) for fathers aged 70 to 74 years, 692 births (0.002%) for fathers aged 75 to 79 years, and 265 births (0.001%) for fathers aged 80 years and older.

**Figure 1.**
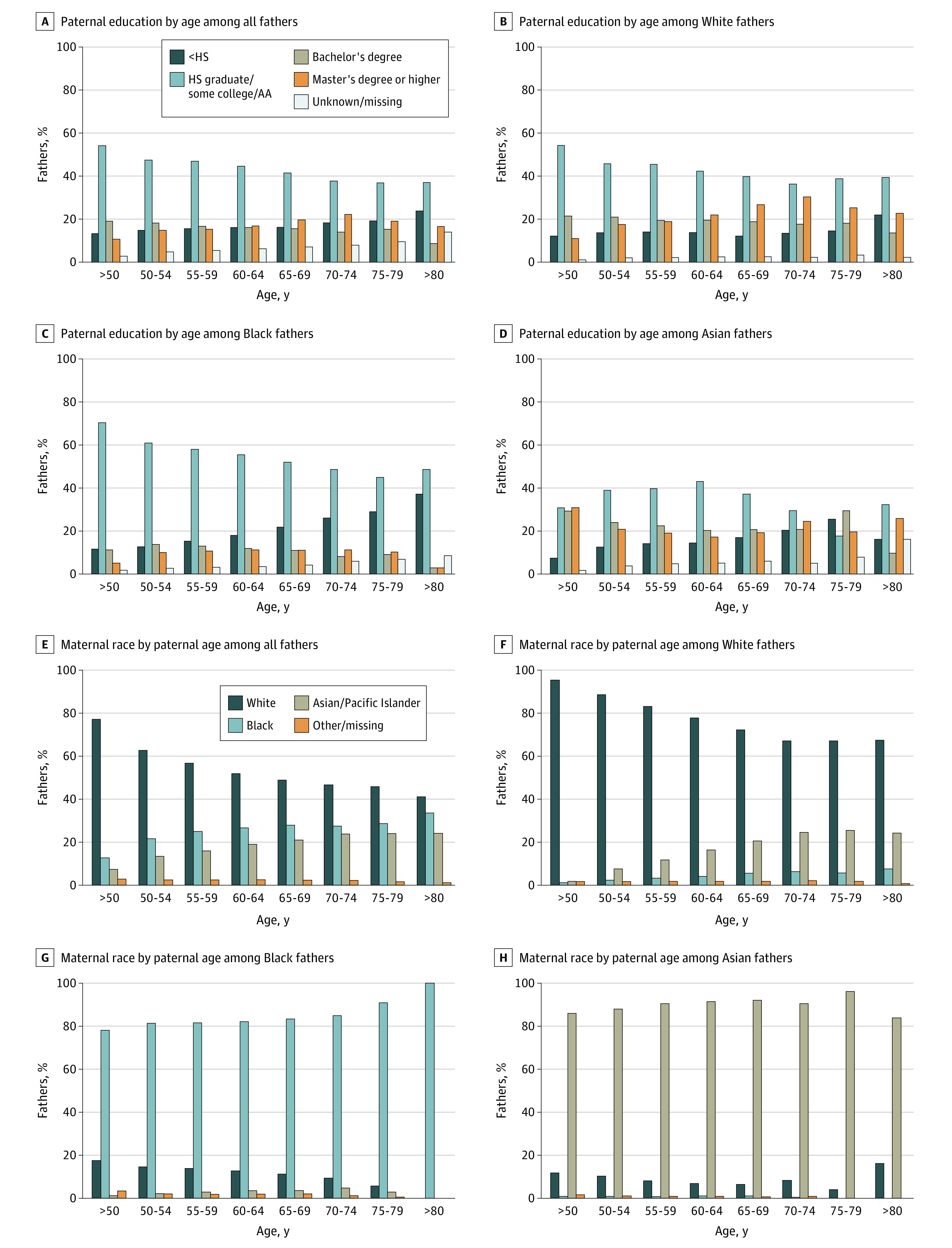
Associations Between Paternal Age and Race with Maternal Race and Paternal Education AA indicates associate’s degree; HS, high school.

As paternal age increased among those aged 50 years and older, a higher proportion identified as Asian or Pacific Islander or Black and a lower proportion as Hispanic. Among fathers younger than 50 years, 2 452 548 were Asian or Pacific Islander (6.3%), 5 270 273 were Black (13.6%), and 8 075 158 were Hispanic (20.9%). In contrast, among fathers 80 years and older, 31 were Asian or Pacific Islander (11.7%), 70 were Black (26.4%), and 27 were Hispanic (10.2%) (Table 1). Education levels also varied by age, with different patterns present based on race and ethnicity (Figure 1A-D; eFigure in Supplement 1). Finally, differences in insurance status were seen among older fathers, with lower utilization of private health insurance in lieu of self-pay and Medicaid with every 5-year increase in paternal age (eTable 5 in Supplement 1).

**Figure 2.**
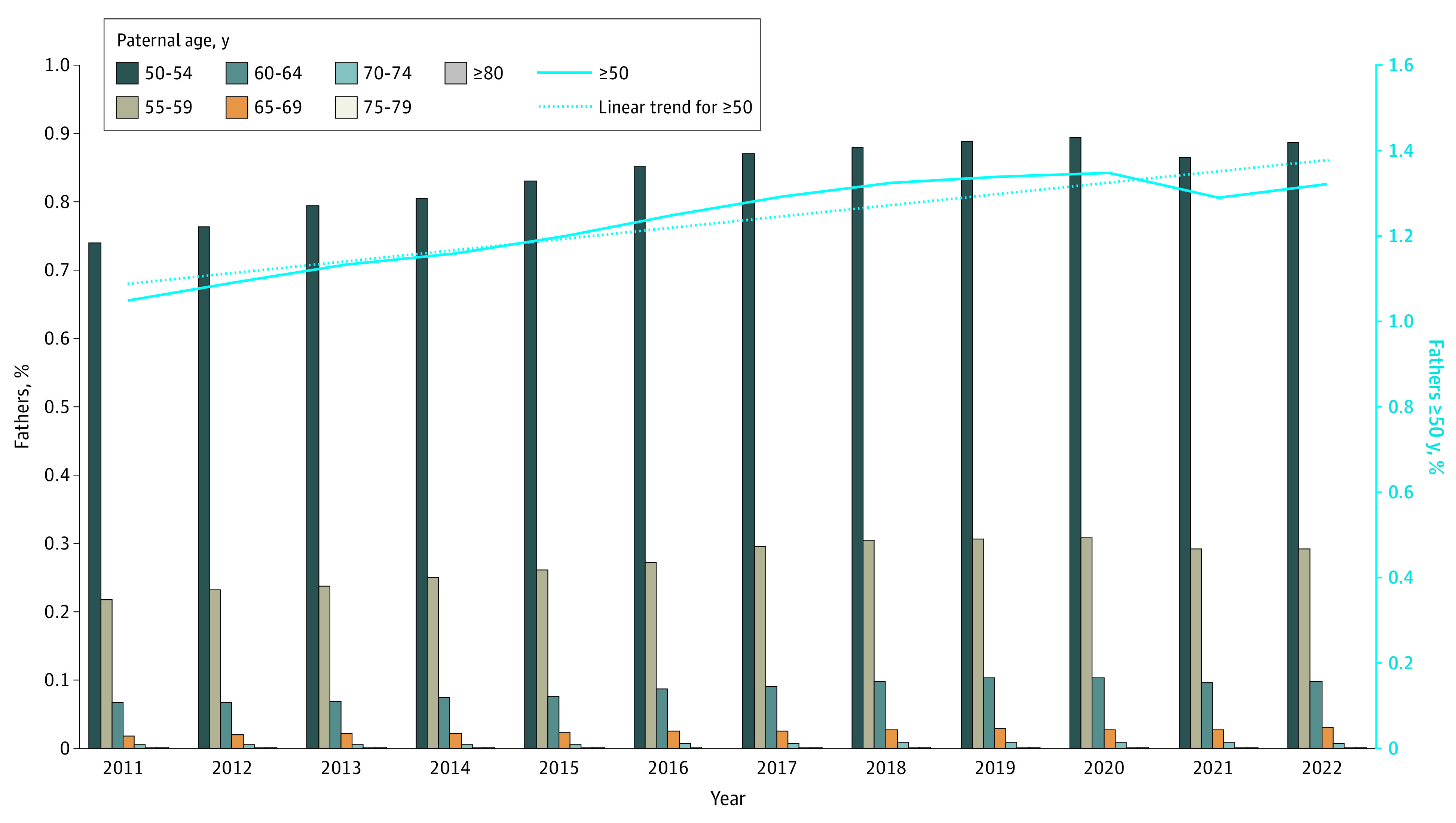
Trends in Older Fatherhood in the United States, 2011-2022

Figure 2 illustrates the proportional increase in APA fathers over time. In 2011, fathers aged ≥50 years accounted for 1.1% of all US births, increasing to 1.3% by 2022 (*P* for trend < .001). The group aged 50 to 54 years had the largest increase, with births increasing from 0.7% in 2011 to 0.9% in 2022 (eTable 6 in Supplement 1). In contrast, the group aged 80 years and older, which had the fewest births overall, documented 16 births in 2011 and 29 births in 2022 (eTable 6 in Supplement 1). A sensitivity analysis excluding 2 501 550 births associated with ART found that 1.0% of births in 2011 occurred among fathers 50 years and older, which significantly increased to 1.2% in 2022 (P for trend < .001).

Significant associations between paternal age and maternal sociodemographic characteristics were observed (Table 1). With advancing paternal age, maternal partners tended to be older, with most in the 31 to 40 years and 41 to 50 years ranges, and a decline in mothers younger than 20 years. Mothers associated with older fathers were more racially diverse compared with those with younger fathers (Table 1 and Figure 1E-H). Additional stratification by paternal race showed divergent preferences for maternal partners. Notably, older White fathers were more frequently partnered with mothers who were Asian or Pacific Islander, Black, or other or unknown race compared with younger fathers (Figure 1E-H). Similarly, stratification by maternal education also showed differences by paternal race and age (Supplement 2).

### Multivariate Analyses

Multivariable logistic regression demonstrated a stepwise association between adverse perinatal outcomes and each 10-year increase in APA (Table 2). Fathers aged 50 to 59 years were associated with a 16% increased risk of preterm birth (adjusted odds ratio [aOR], 1.16; 95% CI, 1.15-1.18) and a 14% increased risk of low birth weight (aOR, 1.14; 95% CI, 1.13-1.15) compared with fathers aged 30 to 39 years. This group also experienced a 13% increase in gestational diabetes (aOR, 1.13; 95% CI, 1.11-1.14). These risks increased with paternal age, with a 21% increased risk of preterm birth (aOR, 1.21; 95% CI, 1.10-1.33) and 24% increased risk in low birth weight (aOR, 1.24; 95% CI 1.12-1.38) in low birthweight for fathers aged 70 years and older. While the fathers aged 60 to 69 years showed increased risks of maternal gestational hypertension and diabetes, these associations were not evident in older age groups.

**Table 2.** Logistic Regression Analysis of Odds of Perinatal Outcomes by Paternal Age

Paternal age, y	OR (95% CI)	
	Unadjusted	Adjusted
Preterm birth (<37 vs ≥37 wk)^a^		
<30	1.04 (1.04-1.04)	1.00 (1.00-1.00)
30-39	1 [Reference]	1 [Reference]
40-49	1.26 (1.26-1.26)	1.09 (1.09-1.09)
50-59	1.51 (1.50-1.52)	1.16 (1.15-1.18)
60-69	1.73 (1.69-1.77)	1.27 (1.24-1.31)
≥70	1.77 (1.63-1.93)	1.21 (1.10-1.33)
Low birth weight (<2500 vs ≥2500g)^a^		
<30	1.09 (1.08-1.09)	1.01 (1.01-1.02)
30-39	1 [Reference]	1 [Reference]
40-49	1.23 (1.23-1.24)	1.06 (1.06-1.07)
50-59	1.53 (1.52-1.55)	1.14 (1.13-1.15)
60-69	1.79 (1.74-1.84)	1.22 (1.19-1.26)
≥70	1.97 (1.79-2.16)	1.24 (1.12-1.38)
Assisted reproductive technology^b^		
<30	0.20 (0.20-0.21)	0.66 (0.65-0.67)
30-39	1 [Reference]	1 [Reference]
40-49	1.85 (1.84-1.86)	1.47 (1.46-1.48)
50-59	2.42 (2.39-2.45)	2.23 (2.19-2.27)
60-69	3.22 (3.10-3.34)	3.50 (3.34-3.65)
≥70	5.49 (4.96-6.08)	6.51 (5.73-7.39)
Gestational diabetes^c^		
<30	0.58 (0.58-0.59)	0.84 (0.84-0.84)
30-39	1 [Reference]	1 [Reference]
40-49	1.38 (1.38-1.39)	1.12 (1.11-1.12)
50-59	1.45 (1.44-1.47)	1.13 (1.11-1.14)
60-69	1.43 (1.39-1.48)	1.09 (1.06-1.13)
≥70	1.43 (1.28-1.59)	1.10 (0.98-1.24)
Gestational hypertension^d^		
<30	1.04 (1.04-1.05)	1.10 (1.10-1.11)
30-39	1 [Reference]	1 [Reference]
40-49	1.08 (1.07-1.08)	0.99 (0.98-0.99)
50-59	1.12 (1.10-1.13)	1.02 (1.01-1.04)
60-69	1.15 (1.10-1.19)	1.11 (1.07-1.16)
≥70	1.05 (0.92-1.20)	1.05 (0.91-1.21)
First maternal birth^a^		
<30	2.20 (2.19-2.20)	1.66 (1.65-1.66)
30-39	1 [Reference]	1 [Reference]
40-49	0.71 (0.70-0.71)	0.90 (0.90-0.91)
50-59	0.79 (0.78-0.79)	1.16 (1.15-1.17)
60-69	0.92 (0.90-0.94)	1.39 (1.36-1.43)
≥70	0.98 (0.91-1.05)	1.55 (1.43-1.69)

Abbreviation: OR, odds ratio.

^a^ Preterm birth, low birth weight, and first maternal birth were adjusted for maternal age, maternal race, parental education, maternal body mass index, insurance status, marital status, smoking during pregnancy, prior preterm birth, and assisted reproductive technology.

^b^ Assisted reproductive technology was adjusted for maternal age, maternal race, maternal education, maternal body mass index, paternal education, insurance status, marital status, smoking during pregnancy, and prior preterm birth.

^c^ Gestational diabetes was adjusted for maternal age, maternal race, maternal education, body mass index, paternal education, insurance status, marital status, smoking during pregnancy, prior preterm birth, assisted reproductive technology, gestational hypertension, and hypertension.

^d^ Gestational hypertension was adjusted for maternal age, maternal race, maternal education, maternal body mass index, paternal education, insurance status, marital status, smoking during pregnancy, prior preterm birth, assisted reproductive technology, gestational diabetes, and diabetes.

Older fathers also demonstrated a higher propensity to use ART and father children with primiparous women. For example, fathers aged 50 to 59 years were more than twice as likely to use ART (aOR, 2.23; 95% CI, 2.19-2.27) and had a 16% increased likelihood of fathering a child with a first-time mother (aOR, 1.16; 95% CI, 1.15-1.17). These associations increased with advancing paternal age: fathers aged 70 years and older were more than 6 times more likely to use ART (aOR, 6.51; 95% CI, 5.73-7.39) and 55% more likely to have children with primiparous women (aOR, 1.55; 95% CI, 1.43-1.69) than those aged 30 to 39 years.

Further stratification by maternal age consistently demonstrated increased odds of preterm birth, low birth weight, and use of ART with APA. However, the likelihood of older fathers having children with first-time mothers was higher only among mothers aged 25 years and older. No consistent association between maternal and paternal age was observed for gestational hypertension and diabetes. (eTable 7 in Supplement 1).

### Infant Sex Ratio

From 2011 to 2022, more male births (23 631 713 births [51.2%]) than female births (22 563 740 births [48.8%]) were observed. While the male-to-female birth ratio was greater than 1 among most APA fathers, fathers aged 70 years and older exhibited an 8% reduction in likelihood of fathering a male child (aOR, 0.92; 95% CI, 0.86-0.99), while fathers aged 75 years and older experienced a 16% decline (aOR, 0.84; 95% CI, 0.73-0.97) (Table 3).

**Table 3.** Association of Paternal Age With Infant Sex Ratio

Paternal age, y (n = 46 195 453)^a^	Births, No. (%)		Male births, OR (95% CI)	
	Female (n = 22 563 740)	Male (n = 23 631 713	Unadjusted	Adjusted^b^
<50	18 894 872 (48.8)	19 832 970 (51.2)	1 [Reference]	1 [Reference]
≥50	237 306 (49.0)	247 201 (51.0)	0.99 (0.98-1.00)	0.99 (0.99-1.00)
<55	19 056 204 (48.8)	20 001 192 (51.2)	1 [Reference]	1 [Reference]
≥55	75 974 (49.0)	78 979 (51.0)	0.99 (0.98-1.00)	0.99 (0.98-1.00)
<60	19 108 764 (48.8)	20 055 800 (51.2)	1 [Reference]	1 [Reference]
≥60	23 414 (49.0)	24 371 (51.0)	0.99 (0.97-1.01)	0.99 (0.97-1.01)
<65	19 125 396 (48.8)	20 073 168 (51.2)	1 [Reference]	1 [Reference]
≥65	6782 (49.2)	7003 (50.8)	0.98 (0.94-1.01)	0.97 (0.93-1.01)
<70	19 130 278 (48.8)	20 078 294 (51.2)	1 [Reference]	1 [Reference]
≥70	1900 (50.3)	1877 (49.7)	0.94 (0.88-1.00)	0.92 (0.86-0.99)
<75	19 131 677 (48.8)	20 079 715 (51.2)	1 [Reference]	1 [Reference]
≥75	501 (52.4)	456 (47.6)	0.88 (0.77-1.00)	0.84 (0.73-0.97)

Abbreviation: OR, odds ratio.

^a^ A total of 3 431 562 female and 3 551 542 male births were excluded due to missing paternal age.

^b^ Adjusted for maternal age, maternal race, parental education, insurance status, marital status, assisted reproductive technology, gestational age, prior preterm birth, and low birth weight. inverse probability weights were used to estimate missing paternal age.

## Discussion

Our cross-sectional study of the entire US birth population from 2011 to 2022 found a significant increase in fathers aged 50 years or older, with nearly 500 000 children conceived by these fathers. Differences in education, marital status, and race were apparent compared with younger US fathers. APA was consistently associated with higher risk of preterm birth and low birth weight despite controlling for maternal factors, with most adverse outcomes occurring after age 50 years as compared with age 30 to 39 years. Importantly, the use of ART was high among this population (>15% for some age groups), implying a significant reliance on this technology. While the overall birth cohort demonstrated a greater percentage of male births, the oldest fathers were significantly less likely to father male children.

The implications of APA have increasingly gained recognition. Male fertility decreases with age, compounded by lifestyle and environmental influences that affect hormonal profiles and erectile function.^10,38,39^ This can lead to diminished sperm quality, characterized by increased DNA fragmentation and increased risk of genetic and epigenetic anomalies transmissible to offspring.^18,20,40,41,42^ Multiple studies have linked paternal age with adverse outcomes, such as congenital anomalies, pediatric cancers, and perinatal outcomes, like preterm birth and low birth weight.^9,21,43,44,45,46^ APA is also associated with decreased fertilization, pregnancy, and live birth rates, necessitating a greater reliance on ART.^28,47,48^ Our findings corroborate these trends in a large contemporary cohort, revealing a small but significant increase in risks of preterm birth, low birth weight, and use of ART among older fathers.

Recent studies have also challenged the stereotype of the older father as uniformly affluent and highly educated, revealing the complex portrait of this demographic.^49,50^ Scandinavian research has found that older fathers come from diverse urban and rural settings, with varied education levels, marital status, and health literacy.^28,29,30^ The trend toward delayed fatherhood is often attributed to diminished concerns of the male “biological clock” and the desire for educational and financial stability prior to starting a family. Previous studies have also highlighted the influence of evolving gender norms that promote active parental involvement while emphasizing traditional roles like the male “breadwinner.”^51,52,53^

Our results highlight the racial and educational heterogeneity among mothers, with a notable increase in maternal primiparity among women aged 25 years and older with APA fathers. Prior studies have also highlighted such gender-specific reproductive behaviors. Kreyenfeld et al^54^ observed that in economically unstable times, highly educated women tended to delay childbearing, while those with less education increasingly became mothers and housewives. In contrast, Swedish researchers suggest that female unemployment did not influence childbearing practices when accounting for economic fluctuations.^55,56^ While paternal primiparity was not available in this analysis, the observed patterns in maternal primiparity with older fathers may be influenced by a multitude of socioeconomic and cultural factors, including the portrayal of older fathers as stable and resourced.^8,57^ It is likely that a combination of personal ambition, cultural shifts, and structural challenges not only prompt men to enter fatherhood in older age but also influence female reproductive habits.

We observed that the oldest fathers were significantly less likely to father male children. The phenomenon of the infant sex ratio, traditionally skewed toward male births, continues to garner attention. Prior studies have focused on the Trivers-Willard hypothesis, which posits the reproductive benefits of siring males from healthy mothers in stable environments and the impact of modulating factors, like environmental pollutants, maternal stress, parity, and paternal age, on the sex ratio.^58,59,60,61,62^ In contrast, Orzack et al^63^ argue against the existence of such a ratio, suggesting a male-biased sex ratio among aborted (abnormal) embryos yet a higher overall female mortality rate during pregnancy to negate any chance of bias. Birth data from 19th century Germany support the male-biased sex ratio, revealing the highest ratio of male-biased births in the most fertile families and the lowest ratio in the least fertile families.^62,64^ Considering the complex web of genetic and environmental influences on this ratio,^65^ further research is imperative.

Overall, delayed fatherhood has significant implications for public health. Certain advantages of having older fathers may include better parenting skills, financial stability, improved nutritional and educational opportunities, and an emotionally richer family life.^50,66^ However, drawbacks, such as social stigma, parental fatigue, increased ART use, and potential negative health outcomes for children, also exist.^67^ In particular, the typically shorter male lifespan raises the prospect of children becoming early caregivers or experiencing the trauma of a parental death.^66,68,69^

### Strengths and Limitations

Several strengths and limitations warrant mention. Our data represent all live US births from 2011 to 2022, providing the most comprehensive assessment of the characteristics of contemporary family building. While missing paternal data have previously been associated with underlying socioeconomic disparities, we used inverse probability weighting to reduce overrepresentation of certain demographics, particularly among older, more educated fathers.^7,21^ However, our maternal-level birth certificate data may introduce potential bias, especially regarding multiparity among partners at the paternal level. Despite our efforts to control for maternal, perinatal, and sociodemographic factors, unmeasured confounding cannot be entirely excluded.

## Conclusions

In this cross-sectional study of all US births from 2011 to 2022, we identified the evolving profile and increase of APA in the US, revealing differences in race and education among these fathers and their partners. The modest but significant associations between APA with adverse perinatal outcomes support existing trends and highlighted the complexities associated with delayed fatherhood. Moreover, a higher proportion of births to older fathers included ART. These ongoing societal shifts underscore the need for public education and further research into the benefits and drawbacks of delayed fatherhood.

## Notes

Supplement 1. **eTable 1.** Parental Race Variables Description and Construction **eTable 2.** Parental Education Variables Description and Construction **eTable 3.** Parental Hispanic Ethnicity Variables Description and Construction **eTable 4.** Additional Sociodemographic Data on Fathers Stratified by Race **eTable 5.** Additional Maternal and Perinatal Characteristics of US Mothers, 2011-2022 **eTable 6.** Number and Percentage of Births Attributed to the Oldest US Fathers by Birth Year **eTable 7.** Association of Paternal Age With Perinatal Outcomes, Stratified by Maternal Age

Supplement 2.
Mother's Education vs Paternal Race and Age

Supplement 3.
Data Sharing Statement
