# Supplementary material for: Sociodemographic Trends and Perinatal Outcomes in Fathers 50 Years and Older
Source: JAMA Netw Open. 2024 Aug 1;7(8):e2425269. doi: 10.1001/jamanetworkopen.2024.25269 (PMC11294967; doi:10.1001/jamanetworkopen.2024.25269)
Supplement: Supplement 1. — eTable 1. Parental Race Variables Description and Construction eTable 2. Parental Education Variables Description and Construction eTable 3. Parental Hispanic Ethnicity Variables Description and Construction eTable 4. Additional Sociodemographic Data on Fathers Stratified by Race eTable 5. Additional Maternal and Perinatal Characteristics of US Mothers, 2011-2022 eTable 6. Number and Percentage of Births Attributed to the Oldest US Fathers by Birth Year eTable 7. Association of Paternal Age With Perinatal Outcomes, Stratified by Maternal Age [file jamanetwopen-e2425269-s001.pdf]

Supplemental Online Content

Ha AS, Scott M, Zhang CA, et al. Sociodemographic trends and perinatal outcomes in fathers 50 years and older. JAMA Netw Open. 2024;7(8):e2425269. doi:10.1001/jamanetworkopen.2024.25269

eTable 1. Parental Race Variables Description and Construction
eTable 2. Parental Education Variables Description and Construction
eTable 3. Parental Hispanic Ethnicity Variables Description and Construction
eTable 4. Additional Sociodemographic Data on Fathers Stratified by Race
eTable 5. Additional Maternal and Perinatal Characteristics of US Mothers, 2011-2022
eTable 6. Number and Percentage of Births Attributed to the Oldest US Fathers by Birth Year
eTable 7. Association of Paternal Age With Perinatal Outcomes, Stratified by Maternal Age
eFigure. Marital Status and Maternal Education by Paternal Race and Age

This supplemental material has been provided by the authors to give readers additional information about their work.

© 2024 Ha AS et al. *JAMA Network Open.*

eTable 1: Parental Race Variables Description and Construction

Maternal Race						
**Covariate**	**2011-2013**		**2014-2022**		**2011-2022**	
	**Code**	**Description**	**Code**	**Description**	**Code**	**Description**
**MRACEREC**	1	White				
	2	Black				
	3	American Indian/Alaskan Native				
	4	Asian/Pacific Islander				
**MRACE6**			1	White (only)		
			2	Black (only)		
			3	AIAN (only)		
			4	Asian (only)		
			5	NHOPI (only)		
			6	More than One Race (only)		
**Mother's Race Combined**					1	White
					2	Black
					3	Asian/Pacific Islander[a]
					4	Other/Missing[b]

a Asian/PI (2011-2013) and Asian + NHOPI (2014-2022) combined
b AIAN, More than one race, and missing/unknown classifications combined

Paternal Race						
**Covariate**	**2011-2013**		**2014-2022**		**2011-2022**	
	**Code**	**Description**	**Code**	**Description**	**Code**	**Description**
**FRACEREC**	1	White				
	2	Black				
	3	American Indian/Alaskan Native				
	4	Asian/Pacific Islander				
**FRACE6**			1	White (only)		
			2	Black (only)		
			3	AIAN (only)		
			4	Asian (only)		
			5	NHOPI (only)		
			6	More than One Race (only)		
			9	Unknown/Not Stated		
**Father's Race Combined**					1	White
					2	Black
					3	Asian/Pacific Islander (PI)[a]
					4	Other[b]
					5	Missing

a Asian/PI (2011-2013) and Asian + NHOPI (2014-2022) combined
b AIAN and More than one race combined

© 2024 Ha AS et al. *JAMA Network Open.*

© 2024 Ha AS et al. *JAMA Network Open.*

eTable 2: Parental Education Variables Description and Construction

Paternal/Maternal Education				
**Covariate**	**2011-2022 Code**	**Description**	**2011-2022 Code**	**Description**
**MEDUC (Maternal) FEDUC (Paternal)**	1	8th grade or less	1	Less than High School[a]
	2	9th-12th grade with no diploma		
	3	High School Graduate or GED completed		
	4	Some college credit, but not a degree	2	HS/Some College/AA[b]
	5	Associate degree		
	6	Bachelor's degree	3	Bachelor's Degree
	7	Master's degree	4	Master's or Higher[c]
	8	Doctorate or Professional degree		
	9	Unknown	5	Unknown/Missing[d]

a 1, 2, and 3 from MEDUC combined to create less than high school
b 4 and 5 from MEDUC combined to create HS/Some College/AA
c 7 and 8 from MEDUC combined to create Master's or Higher
d Unknown and Missing data combined

© 2024 Ha AS et al. *JAMA Network Open.*

eTable 3: Parental Hispanic Ethnicity Variables Description and Construction

**Maternal Hispanic Ethnicity**						
**Covariate**	**2011-2013**		**2014-2022**		**2011-2022**	
	**Code**	**Description**	**Code**	**Description**	**Code**	**Description**
**UMHISP**	0	Non-Hispanic				
	1	Mexican				
	2	Puerto Rican				
	3	Cuban				
	4	Central or South American				
	5	Other and Unknown Hispanic				
	9	Origin unknown or not stated				
**MHISP_R**			0	Non-Hispanic		
			1	Mexican		
			2	Puerto Rican		
			3	Cuban		
			4	Central or South American		
			5	Other and Unknown Hispanic		
			9	Origin unknown or not stated		
**Hispanic Ethnicity**					1	No[a]
					2	Yes[b]
					3	Missing[c]
**Paternal Hispanic Ethnicity**						
**Covariate**	**2011-2013**		**2014-2022**		**2011-2022**	
	**Code**	**Description**	**Code**	**Description**	**Code**	**Description**
**UFHISP**	0	Non-Hispanic				
	1	Mexican				
	2	Puerto Rican				
	3	Cuban				
	4	Central or South American				
	5	Other and Unknown Hispanic				
	9	Origin unknown or not stated				
**FHISP_R**			0	Non-Hispanic		
			1	Mexican		
			2	Puerto Rican		
			3	Cuban		
			4	Central or South American		
			5	Other and Unknown Hispanic		
			9	Origin unknown or not stated		
**Hispanic Ethnicity**					1	No[a]
					2	Yes[b]
					3	Missing[c]

a No was comprised of 0 (2011-2013) and 0 (2014-2022)
b Yes was comprised of 1-5 (2011-2013) and 1-5 (2014-2022)
c Missing was comprised of 9 (2011-2013) and 9 (2014-2022)

© 2024 Ha AS et al. *JAMA Network Open.*

eTable 4: Additional Sociodemographic Data on Fathers Stratified by Race

White Fathers, (No (%))										
**Number of Live Births (n)**	**28,267,031**	**987,135**	**27,007,376**	**189,174**	**57,998**	**17,929**	**5,342**	**1,553**	**392**	**132**
**Paternal Age (years)**	**All**	**Missing**	**<50**	**50-54**	**55-59**	**60-64**	**65-69**	**70-74**	**75-79**	**80+**
**Paternal Education**										
Less than High School	3,329,920 (11.8)	11,008 (1.1)	3,281,414 (12.2)	25,927 (13.7)	8,151 (14.1)	2,474 (13.8)	651 (12.1)	209 (13.5)	57 (14.5)	29 (22.0)
HSG/Some College/AA	14,809,404 (52.4)	23,700 (2.4)	14,662,362 (54.3)	86,499 (45.7)	26,368 (45.5)	7,583 (42.3)	2,124 (39.8)	564 (36.3)	152 (38.8)	52 (39.4)
Bachelor's Degree	5,846,143 (20.7)	2,469 (0.3)	5,787,803 (21.4)	39,723 (21.0)	11,279 (19.4)	3,503 (19.5)	1,003 (18.8)	274 (17.6)	71 (18.1)	18 (13.6)
Master's or Higher	3,030,369 (10.7)	1,344 (0.1)	2,978,912 (11.0)	33,198 (17.6)	10,957 (18.9)	3,932 (21.9)	1,426 (26.7)	471 (30.3)	99 (25.3)	30 (22.7)
Missing	1,251,195 (4.4)	948,614 (96.1)	296,885 (1.1)	3,827 (2.0)	1,243 (2.1)	437 (2.5)	138 (2.6)	35 (2.3)	13 (3.3)	3 (2.3)
**Maternal Education**										
Less than High School	2,826,411 (10.0)	14,053 (1.4)	2,775,497 (10.2)	25,062 (13.2)	8,165 (14.1)	2,564 (14.3)	757 (14.2)	224 (14.4)	64 (16.3)	25 (18.9)
HSG/Some College/AA	13,767,715 (48.7)	31,461 (3.2)	13,617,307 (50.4)	81,731 (43.2)	25,983 (44.8)	7,939 (44.3)	2,359 (44.2)	712 (45.8)	177 (45.2)	46 (34.8)
Bachelor's Degree	6,699,455 (23.7)	3,348 (0.3)	6,631,052 (24.6)	46,208 (24.4)	13,160 (22.7)	4,071 (22.7)	1,175 (22.0)	333 (21.4)	73 (18.6)	35 (26.6)
Master's or Higher	3,857,023 (13.6)	1,688 (0.2)	3,807,638 (14.1)	33,515 (17.8)	9,809 (16.9)	3,069 (17.1)	953 (17.8)	256 (16.6)	70 (17.9)	25 (18.9)
Missing	1,116,427 (4.0)	936,585 (94.9)	175,882 (0.7)	2,658 (1.4)	881 (1.5)	286 (1.6)	98 (1.8)	28 (1.8)	8 (2.0)	1 (0.8)
**Hispanic Status**										
No	21,381,099 (75.6)	746,864 (75.6)	20,429,311 (75.6)	141,263 (74.7)	43,802 (75.5)	13,889 (77.5)	4,230 (79.2)	1,295 (83.4)	330 (84.2)	115 (87.1)
Yes	6,734,502 (23.8)	233,494 (23.7)	6,435,955 (23.8)	46,224 (24.4)	13,621 (23.5)	3,848 (21.4)	1,053 (19.7)	234 (15.1)	57 (14.5)	16 (12.1)
Missing	151,430 (0.6)	6,777 (0.7)	142,110 (0.6)	1,687 (0.9)	575 (1.0)	192 (1.1)	59 (1.1)	24 (1.5)	5 (1.3)	1 (0.8)
**Marital Status**										
Yes	19,621,695 (69.4)	686,891 (69.6)	18,743,316 (69.4)	133,264 (70.4)	40,143 (69.2)	12,659 (70.6)	3,840 (71.9)	1,167 (75.2)	307 (78.3)	108 (81.8)
No	7,002,474 (24.8)	292,936 (29.7)	6,650,890 (24.6)	40,480 (21.4)	13,064 (22.5)	3,742 (20.9)	1,046 (19.6)	258 (16.6)	44 (11.2)	14 (10.6)
Missing	1,642,862 (5.8)	7,308 (0.7)	1,613,170 (6.0)	15,430 (8.2)	4,791 (8.3)	1,528 (8.5)	456 (8.5)	128 (8.2)	41 (10.5)	10 (7.6)

© 2024 Ha AS et al. *JAMA Network Open.*

**Black Fathers**										
**Number of Live Births (n)**	**5,609,342**	**223,315**	**5,270,273**	**75,129**	**27,812**	**9,169**	**2,676**	**722**	**176**	**70**
**Paternal Age (years)**	**All**	**Missing**	**<50**	**50-54**	**55-59**	**60-64**	**65-69**	**70-74**	**75-79**	**80+**
**Paternal Education**										
Less than High School	631,952 (11.3)	4,490 (2.0)	611,173 (11.6)	9,535 (12.7)	4,256 (15.3)	1,650 (18.0)	583 (21.8)	188 (26.0)	51 (29.0)	26 (37.1)
HSG/Some College/AA	3,787,795 (67.5)	14,628 (6.6)	3,704,408 (70.3)	45,715 (60.8)	16,110 (57.9)	5,079 (55.4)	1391 (52.0)	351 (48.6)	79 (44.9)	34 (48.5)
Bachelor's Degree	609,766 (10.9)	1,031 (0.5)	593,314 (11.3)	10,353 (13.8)	3,603 (13.0)	1,095 (11.9)	293 (10.9)	59 (8.2)	16 (9.1)	2 (2.9)
Master's or Higher	281,484 (5.0)	382 (0.2)	269,197 (5.1)	7,516 (10.0)	2,965 (10.6)	1,026 (11.2)	297 (11.1)	81 (11.2)	18 (10.2)	2 (2.9)
Missing	298,345 (5.3)	202,784 (90.7)	92,181 (1.7)	2,010 (2.7)	878 (3.2)	319 (3.5)	112 (4.2)	43 (6.0)	12 (6.8)	6 (8.6)
**Maternal Education**										
Less than High School	615,640 (11.0)	6,360 (2.8)	588,796 (11.2)	12,210 (16.2)	5,316 (19.2)	1,993 (21.7)	666 (24.9)	218 (30.2)	53 (30.1)	28 (40.0)
HSG/Some College/AA	3,610,801 (64.4)	18,416 (8.3)	3,526,247 (66.9)	42,943 (57.2)	15,918 (57.2)	5,254 (57.3)	1,514 (56.6)	376 (52.1)	95 (54.0)	38 (54.3)
Bachelor's Degree	742,818 (13.2)	1,556 (0.7)	724,139 (13.7)	11,663 (15.5)	3,899 (14.0)	1,167 (12.7)	306 (11.4)	74 (10.2)	13 (7.4)	1 (1.4)
Master's or Higher	404,717 (7.2)	537 (0.2)	393,705 (7.5)	7,334 (9.8)	2,307 (8.3)	627 (6.8)	161 (6.0)	37 (5.1)	7 (4.0)	2 (2.9)
Missing	235,366 (4.2)	196,446 (88.0)	37,386 (0.7)	979 (1.3)	372 (1.3)	128 (1.5)	29 (1.1)	17 (2.4)	8 (4.5)	1 (1.4)
**Hispanic Status**										
No	5,348,781 (95.4)	206,658 (92.5)	5,030,674 (95.5)	72,469 (96.5)	26,668 (95.9)	8,822 (96.2)	2556 (95.5)	692 (95.8)	175 (99.4)	67 (95.7)
Yes	211,508 (3.8)	14,309 (6.4)	194,502 (3.7)	169 (2.3)	697 (2.5)	216 (2.4)	67 (2.5)	16 (2.3)	1 (0.6)	1 (1.4)
Missing	49,053 (0.9)	2,348 (1.1)	45,097 (0.8)	961 (1.3)	447 (1.6)	131 (1.4)	53 (2.0)	14 (1.9)	0 (0.0)	2 (2.9)
**Marital Status**										
Yes	2,189,726 (39.0)	80,517 (36.1)	2,044,381 (38.8)	42,203 (56.2)	15,481 (55.7)	4,995 (54.5)	1,560 (58.3)	438 (60.7)	109 (61.9)	42 (60.0)
No	3,269,701 (58.3)	139,853 (62.6)	3,083,084 (58.5)	30,359 (40.4)	11,217 (40.3)	3,807 (41.5)	1,035 (38.7)	258 (35.7)	61 (34.7)	27 (38.6)
Missing	149,915 (2.7)	2,945 (1.3)	142,808 (2.7)	2,567 (3.4)	1,114 (4.0)	367 (4.0)	81 (3.0)	26 (3.6)	6 (3.4)	1 (1.4)

© 2024 Ha AS et al. *JAMA Network Open.*

© 2024 Ha AS et al. *JAMA Network Open.*

Asian Fathers										
**Number of Live Births (n)**	**2,601,655**	**106,487**	**2,452,548**	**29,051**	**9,322**	**3,071**	**853**	**241**	**51**	**31**
**Paternal Age (years)**	**All**	**Missing**	**<50**	**50-54**	**55-59**	**60-64**	**65-69**	**70-74**	**75-79**	**80+**
**Paternal Education**										
Less than High School	187,237 (7.2)	552 (0.5)	181,074 (7.4)	3,638 (12.5)	1,317 (14.1)	444 (14.5)	145 (17.0)	49 (20.3)	13 (25.5)	5 (16.1)
HSG/Some College/AA	772,209 (29.7)	1,264 (1.2)	754,203 (30.8)	11,317 (39.0)	3,699 (39.7)	1,319 (43.0)	317 (37.2)	71 (29.5)	9 (17.6)	10 (32.3)
Bachelor's Degree	728,171 (28.0)	618 (0.6)	717,646 (29.3)	6,953 (23.9)	2,087 (22.4)	623 (20.3)	176 (20.6)	50 (20.7)	15 (29.4)	3 (9.7)
Master's or Higher	766,661 (29.4)	523 (0.5)	757,543 (30.8)	6,049 (20.8)	1,777 (19.1)	528 (17.1)	164 (19.2)	59 (24.5)	10 (19.6)	8 (25.8)
Missing	147,377 (5.7)	103,530 (97.2)	42,082 (1.7)	1,094 (3.8)	442 (4.7)	157 (5.1)	51 (6.0)	12 (5.0)	4 (7.9)	5 (16.1)
**Maternal Education**										
Less than High School	191,455 (7.3)	733 (0.7)	183,536 (7.5)	4,515 (15.5)	1,719 (18.5)	666 (21.7)	201 (23.6)	64 (26.6)	12 (23.5)	9 (29.0)
HSG/Some College/AA	763,338 (29.3)	1728 (1.6)	744,610 (30.4)	11,470 (39.5)	3,788 (40.6)	1,272 (41.4)	344 (40.3)	92 (38.2)	22 (43.2)	12 (38.7)
Bachelor's Degree	805,609 (31.0)	567 (0.5)	794,281 (32.4)	7,611 (26.2)	2,222 (23.8)	680 (22.1)	197 (23.1)	43 (17.8)	6 (11.8)	2 (6.5)
Master's or Higher	698,975 (26.9)	311 (0.3)	692,805 (28.2)	4,317 (14.9)	1,155 (12.4)	275 (9.0)	72 (8.4)	26 (10.8)	9 (17.6)	5 (16.1)
Missing	142,278 (5.5)	103,148 (96.9)	37,316 (1.5)	1,138 (3.9)	438 (4.7)	178 (5.8)	39 (4.6)	16 (6.6)	2 (3.9)	3 (9.7)
**Hispanic Status**										
No	2,545,028 (97.8)	101,186 (95.0)	2,402,017 (97.9)	28,526 (98.2)	9,139 (98.0)	3,009 (98.0)	833 (97.7)	237 (98.4)	50 (98.0)	31 (100.0)
Yes	44,002 (1.7)	4,746 (4.5)	38,826 (1.6)	279 (1.0)	109 (1.2)	31 (1.0)	9 (1.0)	2 (0.8)	0 (0.0)	0 (0.0)
Missing	12,625 (0.5)	555 (0.5)	11,705 (0.5)	246 (0.8)	74 (0.8)	31 (1.0)	11 (1.3)	2 (0.8)	1 (2.0)	0 (0.0)
**Marital Status**										
Yes	1,993,607 (76.6)	84,893 (79.7)	1,877,525 (76.6)	21,365 (73.5)	6,753 (72.5)	2,226 (72.5)	610 (71.5)	177 (73.4)	37 (72.5)	21 (67.7)
No	264,455 (10.2)	20,633 (19.4)	240,279 (9.8)	2,475 (8.6)	777 (8.3)	211 (6.9)	67 (7.9)	10 (4.2)	1 (2.0)	2 (6.5)
Missing	343,593 (13.2)	961 (0.9)	334,744 (13.6)	5,211 (17.9)	1792 (19.2)	634 (20.6)	176 (20.6)	54 (22.4)	13 (25.5)	8 (25.8)

© 2024 Ha AS et al. *JAMA Network Open.*

eTable 5: Additional Maternal and Perinatal Characteristics of US Mothers (2011-2022)

**Number of Live Births (N)**	**46,195,453**	**6,983,104**	**38,727,842**	**329,554**	**107,168**	**34,000**	**10,008**	**2,820**	**692**	**265**
**Paternal Age (years)**	**All**	**Missing**	**<50**	**50-54**	**55-59**	**60-64**	**65-69**	**70-74**	**75-79**	**80+**
Maternal Characteristics, %										
**BMI, (kg/m^2)**										
Underweight (<18.5)	1,449,633 (3.1)	225,435 (3.2)	1,208,257 (3.1)	10,199 (3.1)	3,818 (3.5)	1,338 (3.9)	421 (4.2)	129 (4.6)	26 (3.7)	10 (3.8)
Normal (18.5-24.9)	18,718,787 (40.5)	1,976,795 (28.3)	16,545,228 (42.7)	132,659 (40.3)	43,917 (41.0)	14,183 (41.7)	4,337 (43.3)	1,243 (44.0)	301 (43.5)	124 (46.8)
Overweight (25.0-29.9)	11,388,164 (24.7)	1,290,675 (18.5)	9,970,181 (25.7)	86,960 (26.4)	27,963 (26.1)	8,896 (26.2)	2,546 (25.5)	710 (25.2)	179 (25.9)	54 (20.4)
Obese (30.0-39.9)	94,86,366 (20.5)	1,268,471 (18.2)	8,114,053 (21.0)	71,898 (21.8)	22,561 (21.1)	6755 (19.9)	1,941 (19.4)	499 (17.7)	130 (18.8)	58 (21.9)
Morbid Obesity (40.0+)	3,536,374 (7.7)	605,599 (8.7)	2,890,123 (7.5)	27,838 (8.4)	8,909 (8.3)	2,828 (8.3)	763 (7.6)	239 (8.5)	56 (8.1)	19 (7.1)
Missing	1,616,129 (3.5)	1,616,129 (23.1)	0 (0.0)	0 (0.0)	0 (0.0)	0 (0.0)	0 (0.0)	0 (0.0)	0 (0.0)	0 (0.0)
**Insurance Status**										
Medicaid	18,815,838 (40.7)	3,985,218 (57.1)	14,634,610 (37.8)	130,128 (39.5)	45,273 (42.2)	14,609 (43.0)	4,353 (43.5)	1,210 (42.9)	309 (44.6)	128 (48.4)
Private Insurance	21,693,019 (47.0)	900,556 (12.9)	20,554,856 (53.0)	165,721 (50.3)	50,269 (46.9)	15,521 (45.7)	4,422 (44.2)	1,269 (45.0)	296 (42.8)	109 (41.1)
Self-Pay	1,872,268 (4.0)	229,475 (3.3)	1,612,722 (4.2)	19,864 (6.0)	6,920 (6.5)	2,305 (6.7)	727 (7.3)	190 (6.7)	48 (6.9)	17 (6.4)
Other[a]	1,797,823 (3.9)	190,110 (2.7)	1,591,275 (4.1)	10,858 (3.3)	3,736 (3.5)	1,242 (3.6)	435 (4.3)	126 (4.5)	33 (4.8)	8 (3.0)
Missing	2,016,505 (4.4)	1,677,745 (24.0)	334,379 (0.9)	2,983 (0.9)	970 (0.9)	323 (1.0)	71 (0.7)	25 (0.9)	6 (0.9)	3 (1.1)
**Marital Status**										
Married	26,011,358 (56.3)	1,287,036 (18.4)	24,409,744 (63.0)	215,266 (65.3)	68,308 (63.8)	21,783 (64.0)	6,617 (66.1)	1,926 (68.3)	494 (71.4)	184 (69.4)
Unmarried	17,545,295 (38.0)	5,526,305 (79.2)	11,889,985 (30.7)	86,872 (26.4)	29,607 (27.6)	9,166 (27.0)	2,537 (25.4)	643 (22.8)	125 (18.1)	55 (20.8)
Missing	2,638,800 (5.7)	169,763 (2.4)	2,428,113 (6.3)	27,416 (8.3)	9,253 (8.6)	3,051 (9.0)	854 (8.5)	251 (8.9)	73 (10.5)	26 (9.8)
**Pregnancy Smoking**										
No	40,745,224 (88.2)	4,338,739 (62.1)	35,962,981 (92.9)	302,599 (91.8)	97,376 (90.9)	30,975 (91.1)	9,117 (91.1)	2,564 (90.9)	636 (91.9)	237 (89.4)
Yes	2,989,520 (6.5)	875,452 (12.6)	2,081,240 (5.3)	21,348 (6.5)	8,005 (7.4)	2,488 (7.3)	715 (7.1)	207 (7.3)	41 (5.9)	24 (9.1)

© 2024 Ha AS et al. *JAMA Network Open.*

Missing	2,460,709 (5.3)	1,768,913 (25.3)	683,621 (1.8)	5,607 (1.7)	1,787 (1.7)	537 (1.6)	176 (1.8)	49 (1.8)	15 (2.2)	4 (1.5)
**Number of Live Births (N)**	**46,195,453**	**6,983,104**	**38,727,842**	**329,554**	**107,168**	**34,000**	**10,008**	**2,820**	**692**	**265**
**Paternal Age (years)**	**All**	**Missing**	**<50**	**50-54**	**55-59**	**60-64**	**65-69**	**70-74**	**75-79**	**80+**
Maternal Characteristics, %										
**Hypertension**										
No	43,627,526 (94.4)	5210774 (74.6)	37,950,508 (98.0)	317,259 (96.2)	103,064 (96.2)	32,650 (96.0)	9,624 (96.1)	2,716 (96.3)	672 (97.1)	259 (97.7)
Yes	877,868 (1.9)	139,172 (2.0)	721,222 (1.9)	11,778 (3.6)	3,919 (3.6)	1,285 (3.8)	369 (3.7)	99 (3.5)	18 (2.6)	6 (2.3)
Missing	1,690,059 (3.7)	1,633,158 (23.4)	56,112 (0.1)	517 (0.2)	185 (0.2)	65 (0.2)	15 (0.2)	5 (0.2)	2 (0.3)	0 (0.0)
**Diabetes**										
No	44,102,992 (95.5)	5,293,491 (75.8)	38,334,312 (99.0)	323,419 (98.1)	104,953 (97.9)	33,310 (98.0)	9,792 (97.8)	2,781 (98.6)	675 (97.5)	259 (97.7)
Yes	402,402 (0.9)	56,455 (0.8)	337,418 (0.9)	5,618 (1.7)	2,030 (1.9)	625 (1.8)	201 (2.0)	34 (1.2)	15 (2.2)	6 (2.3)
Missing	1,690,059 (3.5)	1,633,158 (23.4)	56,112 (0.1)	517 (0.2)	185 (0.2)	65 (0.2)	15 (0.2)	5 (0.2)	2 (0.3)	0 (0.0)
**Prior Preterm Birth**										
No	43,096,886 (93.3)	5,108,204 (73.1)	37,524,744 (96.9)	315,720 (95.8)	102,486 (95.6)	32,524 (95.6)	9,579 (95.7)	2,706 (96.1)	667 (96.4)	256 (96.6)
Yes	1,408,508 (3.0)	241,742 (3.5)	1,146,986 (3.0)	13,317 (4.0)	4,497 (4.2)	1,411 (4.2)	414 (4.1)	109 (3.9)	23 (3.3)	9 (3.4)
Missing	1,690,059 (3.7)	1,633,158 (23.4)	56,112 (0.1)	517 (0.2)	185 (0.2)	65 (0.2)	15 (0.2)	5 (0.1)	2 (0.3)	0 (0.0)
Perinatal Characteristics, %										
**Infant Sex**										
Female	22,563,740 (48.8)	3,431,562 (49.1)	18,894,872 (48.8)	161,332 (49.0)	52,560 (49.0)	16,632 (48.9)	4,882 (48.8)	1,399 (49.6)	370 (53.5)	131 (49.4)
Male	23,631,713 (51.2)	3,551,542 (50.9)	19,832,970 (51.2)	168,222 (51.0)	54,608 (51.0)	17,368 (51.1)	51,26 (51.2)	1,421 (50.4)	322 (46.5)	134 (50.6)
**Preterm Birth**										
<37 weeks	5,401,385 (11.7)	1,082,764 (15.5)	4,243,606 (10.9)	49,649 (15.1)	17,149 (16.0)	5,812 (17.1)	1,739 (17.4)	484 (17.2)	133 (19.3)	49 (18.5)
≥37 weeks	40,755,140 (88.2)	5,886,563 (84.3)	34,459,560 (89.0)	279,598 (84.8)	89,907 (83.9)	28,153 (82.8)	8,253 (82.5)	2,332 (82.7)	558 (80.6)	216 (81.5)
Missing	38,928 (0.1)	13,777 (0.2)	24,676 (0.1)	307 (0.1)	112 (0.1)	35 (0.1)	16 (0.1)	4 (0.1)	1 (0.1)	0 (0.0)
**Low Birth Weight**										
≥2500 grams	42,364,329 (91.7)	6,168,016 (88.4)	35,765,142 (92.3)	294,510 (89.4)	94,835 (88.5)	29,826 (87.7)	8,731 (87.3)	2,460 (87.2)	578 (83.5)	231 (87.2)

© 2024 Ha AS et al. *JAMA Network Open.*

<2500 grams	3,787,235 (8.2)	805,128 (11.5)	2,929,229 (7.6)	34,730 (10.5)	12,232 (11.4)	4,147 (12.2)	1,265 (12.6)	357 (12.7)	113 (16.4)	34 (12.8)
Missing	43,889 (0.1)	9,960 (0.1)	33,471 (0.1)	314 (0.1)	101 (0.1)	27 (0.1)	12 (0.1)	3 (0.1)	1 (0.1)	0 (0.0)
**Number of Live Births (N)**	**46,195,453**	**6,983,104**	**38,727,842**	**329,554**	**107,168**	**34,000**	**10,008**	**2,820**	**692**	**265**
**Paternal Age (years)**	**All**	**Missing**	**<50**	**50-54**	**55-59**	**60-64**	**65-69**	**70-74**	**75-79**	**80+**
Perinatal Characteristics, %										
**First Live Birth**										
No	28,213,715 (61.1)	4,144,442 (59.4)	23,719,239 (61.2)	240,210 (72.9)	76,675 (71.6)	23,736 (69.8)	6,828 (68.2)	1,947 (69.0)	468 (67.6)	170 (64.2)
Yes	17,796,236 (38.5)	2,803,477 (40.1)	14,860,557 (38.4)	87,857 (26.7)	29,942 (27.9)	10,092 (29.7)	3,132 (31.3)	868 (30.8)	220 (31.8)	91 (34.3)
Missing	185,502 (0.4)	35,185 (0.5)	148,046 (0.4)	1,487 (0.4)	551 (0.5)	172 (0.5)	48 (0.5)	5 (0.2)	4 (0.6)	4 (1.5)
**ART**										
No	43,693,903 (94.5)	5,314,188 (76.1)	37,927,457 (97.9)	308,823 (93.7)	99,928 (93.2)	31,297 (92.0)	8,960 (89.5)	2,440 (86.5)	580 (83.8)	230 (86.8)
Yes	811,491 (1.8)	35,758 (0.5)	744,273 (2.0)	20,214 (6.1)	7,055 (6.6)	26,38 (7.8)	1,033 (10.3)	375 (13.3)	110 (15.9)	35 (13.2)
Missing	1,690,059 (3.7)	1,633,158 (23.4)	56,112 (0.1)	517 (0.2)	185 (0.2)	65 (0.2)	15 (0.2)	5 (0.2)	2 (0.3)	0 (0.0)

Abbreviations: HSG = High School Graduate; AA = Associate's Degree, ART = Assisted Reproductive Technology
[a]Other Insurance includes: Indian health Service, CHAMPUS/TRICARE, Other Government Insurance, and Other forms of Coverage

© 2024 Ha AS et al. *JAMA Network Open.*

eTable 6: Table Summarizing Number and Percentage of Births Attributed to the Oldest US fathers by Birth Year

**Year**	**2011**	**2012**	**2013**	**2014**	**2015**	**2016**	**2017**	**2018**	**2019**	**2020**	**2021**	**2022**
**Total Births (n)**	2,954,341	3,048,018	3,114,471	3,369,843	3,444,274	3,486,521	3,406,952	3,360,384	3,324,548	3,167,608	3,257,818	3,277,571
**Births[b], No (%))**												
**≥50[a] years old**	**31,019 (1.1)**	**33,288 (1.1)**	**35,308 (1.1)**	**39,106 (1.2)**	**41,297 (1.2)**	**43,518 (1.2)**	**44,045 (1.3)**	**44,450 (1.3)**	**44,531 (1.3)**	**42,656 (1.4)**	**42,024 (1.3)**	**43,265 (1.3)**
50-54 years old	21,844 (0.7)	23,265 (0.8)	24,771 (0.8)	27,143 (0.8)	28,588 (0.8)	29,748 (0.9)	29,633 (0.9))	295,47 (0.9)	29,520 (0.9)	283,12 (0.9)	28,142 (0.9)	29,041 (0.9)
55-59 years old	6,415 (0.2)	7,068 (0.2)	7,411 (0.2)	8,410 (0.3)	8,989 (0.3)	9,503 (0.3)	10,072 (0.3)	10,238 (0.3)	10,173 (0.3)	9,775 (0.3)	9,510 (0.3)	9,604 (0.3)
60-64 years old	1,997 (0.07)	2,074 (0.07)	2,185 (0.07)	2,524 (0.08)	2,646 (0.08)	3,051 (0.088)	3,091 (0.091)	3,321 (0.099)	3,455 (0.1)	3,303 (0.1)	3,118 (0.1)	3,235 (0.1)
65-69 years old	571 (0.02)	650 (0.02)	685 (0.02)	757 (0.02)	796 (0.02)	906 (0.03)	899 (0.03)	924 (0.03)	1006 (0.03)	898 (0.03)	885 (0.03)	1031 (0.03)
70-74 years old	136 (0.005)	177 (0.006)	180 (0.006)	212 (0.006)	210 (0.006)	243 (0.007)	259 (0.008)	306 (0.009)	286 (0.01)	274 (0.01)	279 (0.01)	258 (0.01)
75-79 years old	40 (0.001)	34 (0.001)	54 (0.002)	43 (0.001)	45 (0.001)	54 (0.002)	60 (0.002)	84 (0.002)	70 (0.0)	78 (0.0)	63 (0.0)	67 (0.0)
80+ years old	16 (0.001)	20 (0.001)	22 (0.001)	17 (0.001)	23 (0.001)	13 (0.0)	31 (0.001)	30 (0.001)	21 (0.0)	16 (0.0)	27 (0.0)	29 (0.0)

[a]50 years or older in aggregate
[b]Number of Births Attributed to the Oldest Fathers

eTable 7: The Association of Paternal Age with Perinatal Outcomes, Stratified by Maternal Age

Preterm Birth[a]						
	Mothers <25		Mothers 25-34		Mothers 35+	
Paternal Age (years)	Unadjusted OR (95% CI)	Adjusted OR (95% CI)	Unadjusted OR (95% CI)	Adjusted OR (95% CI)	Unadjusted OR (95% CI)	Adjusted OR (95% CI)
<30	1.02 (1.01, 1.02)	0.99 (0.98, 1.00)	1.04 (1.04, 1.05)	1.03 (1.03, 1.03)	1.32 (1.31, 1.34)	1.07 (1.06, 1.09)
30-39	Ref	Ref	Ref	Ref	Ref	Ref
40-49	1.14 (1.12, 1.16)	1.09 (1.06, 1.11)	1.18 (1.17, 1.19)	1.05 (1.04, 1.05)	1.15 (1.15, 1.16)	1.02 (1.01, 1.02)
50-59	1.24 (1.19, 1.30)	1.16 (1.10, 1.22)	1.40 (1.38, 1.43)	1.15 (1.13, 1.17)	1.40 (1.39, 1.42)	1.07 (1.06, 1.08)
60-69	1.41 (1.25, 1.61)	1.18 (1.03, 1.36)	1.55 (1.48, 1.62)	1.21 (1.15, 1.28)	1.65 (1.60, 1.70)	1.19 (1.15, 1.23)
70+	1.38 (0.95, 2.00)	1.29 (0.85, 1.95)	1.78 (1.53, 2.06)	1.27 (1.07, 1.50)	1.63 (1.46, 1.82)	1.08 (0.96, 1.22)
**Low Birth Weight[a]**						
<30	1.02 (1.01, 1.03)	0.99 (0.98, 1.00)	1.05 (1.04, 1.05)	1.03 (1.03, 1.04)	1.31 (1.29, 1.32)	1.08 (1.07, 1.10)
30-39	Ref	Ref	Ref	Ref	Ref	Ref
40-49	1.14 (1.11, 1.16)	1.05 (1.03, 1.07)	1.18 (1.18, 1.19)	1.03 (1.03, 1.04)	1.15 (1.14, 1.16)	1.01 (1.01, 1.02)
50-59	1.27 (1.20, 1.33)	1.09 (1.03, 1.15)	1.45 (1.42, 1.47)	1.12 (1.09, 1.14)	1.46 (1.44, 1.48)	1.08 (1.06, 1.09)
60-69	1.43 (1.24, 1.65)	1.15 (0.98, 1.34)	1.73 (1.65, 1.83)	1.24 (1.17, 1.31)	1.69 (1.63, 1.75)	1.14 (1.10, 1.19)
70+	1.93 (1.33, 2.80)	1.65 (1.08, 2.51)	2.25 (1.92, 2.63)	1.52 (1.27, 1.81)	1.74 (1.54, 1.98)	1.06 (0.92, 1.22)
**Assisted Reproductive Technology[b]**						
<30	0.67 (0.64, 0.70)	0.86 (0.82, 0.91)	0.46 (0.45, 0.46)	0.80 (0.79, 0.80)	0.30 (0.29, 0.31)	0.69 (0.67, 0.72)
30-39	Ref	Ref	Ref	Ref	Ref	Ref
40-49	1.19 (1.05, 1.33)	1.54 (1.35, 1.77)	1.00 (0.99, 1.02)	1.38 (1.36, 1.40)	1.40 (1.39, 1.41)	1.06 (1.05, 1.07)
50-59	1.85 (1.46, 2.34)	2.54 (1.94, 3.32)	1.09 (1.04, 1.13)	2.07 (1.98, 2.16)	1.94 (1.91, 1.97)	1.45 (1.43, 1.48)
60-69	4.49 (2.89, 6.97)	5.33 (3.15, 9.03)	1.73 (1.57, 1.90)	3.51 (3.15, 3.90)	2.61 (2.50, 2.72)	2.17 (2.06, 2.28)
70+	15.56 (7.70, 31.44)	18.90 (7.64, 46.79)	3.30 (2.60, 4.18)	7.31 (5.59, 9.55)	4.49 (4.00, 5.04)	3.78 (3.26, 4.38)
**Gestational Diabetes[c]**						
<30	0.83 (0.82, 0.84)	0.96 (0.95, 0.98)	0.84 (0.84, 0.84)	0.99 (0.98, 0.99)	1.15 (1.14, 1.17)	0.95 (0.94, 0.97)
30-39	Ref	Ref	Ref	Ref	Ref	Ref
40-49	0.97 (0.94, 1.00)	1.01 (0.97, 1.04)	1.11 (1.10, 1.12)	1.02 (1.01, 1.03)	1.16 (1.16, 1.17)	1.03 (1.02, 1.03)
50-59	0.92 (0.85, 1.00)	0.97 (0.89, 1.07)	1.10 (1.08, 1.13)	1.01 (0.99, 1.04)	1.27 (1.25, 1.28)	1.02 (1.00, 1.03)
60-69	1.03 (0.82, 1.30)	1.11 (0.86, 1.42)	1.04 (0.97, 1.11)	0.93 (0.87, 1.00)	1.30 (1.25, 1.35)	1.00 (0.96, 1.05)
70+	1.23 (0.65, 2.30)	1.48 (0.76, 2.89)	0.99 (0.79, 1.24)	0.84 (0.66, 1.07)	1.40 (1.24, 1.59)	1.07 (0.93, 1.22)
**Gestational Hypertension[d]**						
<30	1.17 (1.16, 1.18)	1.11 (1.10, 1.13)	1.09 (1.08, 1.09)	1.08 (1.08, 1.09)	1.14 (1.12, 1.16)	1.00 (0.99, 1.02)
30-39	Ref	Ref	Ref	Ref	Ref	Ref
40-49	0.98 (0.95, 1.01)	1.00 (0.97, 1.03)	0.95 (0.94, 0.96)	0.96 (0.95, 0.96)	1.05 (1.05, 1.06)	0.96 (0.96, 0.97)
50-59	0.95 (0.88, 1.02)	1.00 (0.93, 1.07)	0.92 (0.90, 0.94)	0.98 (0.95, 1.00)	1.13 (1.12, 1.15)	0.98 (0.96, 0.99)
60-69	1.02 (0.84, 1.24)	1.09 (0.88, 1.35)	0.94 (0.88, 1.01)	1.08 (1.00, 1.17)	1.19 (1.14, 1.24)	1.04 (0.99, 1.09)
70+	1.21 (0.71, 2.05)	1.32 (0.74, 2.36)	0.94 (0.74, 1.19)	1.10 (0.84, 1.42)	1.04 (0.88, 1.23)	0.90 (0.75, 1.08)

© 2024 Ha AS et al. *JAMA Network Open.*

Maternal First Live Birth[a]						
	Mothers <25	Mothers 25-34	Mothers 35+			
**Paternal Age**	**Unadjusted**	**Adjusted**	**Unadjusted**	**Adjusted**	**Unadjusted**	**Adjusted**
**(years)**	**OR (95% CI)**	**OR (95% CI)**	**OR (95% CI)**	**OR (95% CI)**	**OR (95% CI)**	**OR (95% CI)**
<30	1.68 (1.68, 1.69)	1.09 (1.08, 1.09)	1.27 (1.27, 1.27)	1.23 (1.23, 1.24)	0.90 (0.89, 0.91)	1.32 (1.31, 1.34)
30-39	Ref	Ref	Ref	Ref	Ref	Ref
40-49	0.93 (0.92, 0.94)	0.95 (0.94, 0.97)	0.79 (0.79, 0.80)	1.08 (1.08, 1.09)	0.91 (0.91, 0.91)	1.00 (0.99, 1.00)
50-59	0.94 (0.91, 0.97)	0.98 (0.94, 1.01)	0.80 (0.79, 0.81)	1.24 (1.23, 1.26)	1.07 (1.06, 1.08)	1.35 (1.34, 1.37)
60-69	1.14 (1.04, 1.24)	1.15 (1.03, 1.28)	0.87 (0.84, 0.90)	1.39 (1.33, 1.45)	1.27 (1.24, 1.31)	1.70 (1.65, 1.76)
70+	1.05 (0.80, 1.36)	0.90 (0.64, 1.25)	0.88 (0.78, 1.00)	1.50 (1.30, 1.73)	1.39 (1.26, 1.53)	1.99 (1.78, 2.22)

Abbreviations: OR: Odds Ratio; CI: Confidence Interval

[a]Preterm birth, Low Birth Weight, and First Maternal Birth adjusted for: Maternal Age, Maternal Race, Maternal Education, BMI, Paternal Education, Insurance Status, Marital Status, Smoking during Pregnancy, Prior Preterm Birth, and ART

[b]Assisted Reproductive Technology (ART) adjusted for: Maternal Age, Maternal Race, Maternal Education, BMI, Paternal Education, Insurance Status, Marital Status, Smoking during Pregnancy, and Prior Preterm Birth

[c]Gestational Diabetes adjusted for: Maternal Age, Maternal Race, Maternal Education, BMI, Paternal Education, Insurance Status, Marital Status, Smoking during Pregnancy, Prior Preterm Birth, Gestational hypertension, and hypertension

[d]Gestational Hypertension adjusted for: Maternal Age, Maternal Race, Maternal Education, BMI, Paternal Education, Insurance Status, Marital Status, Smoking during Pregnancy, Prior Preterm Birth, Gestational diabetes, and diabetes

© 2024 Ha AS et al. *JAMA Network Open.*

eFigure 1: Marital Status and Maternal Education by Paternal Race and Age

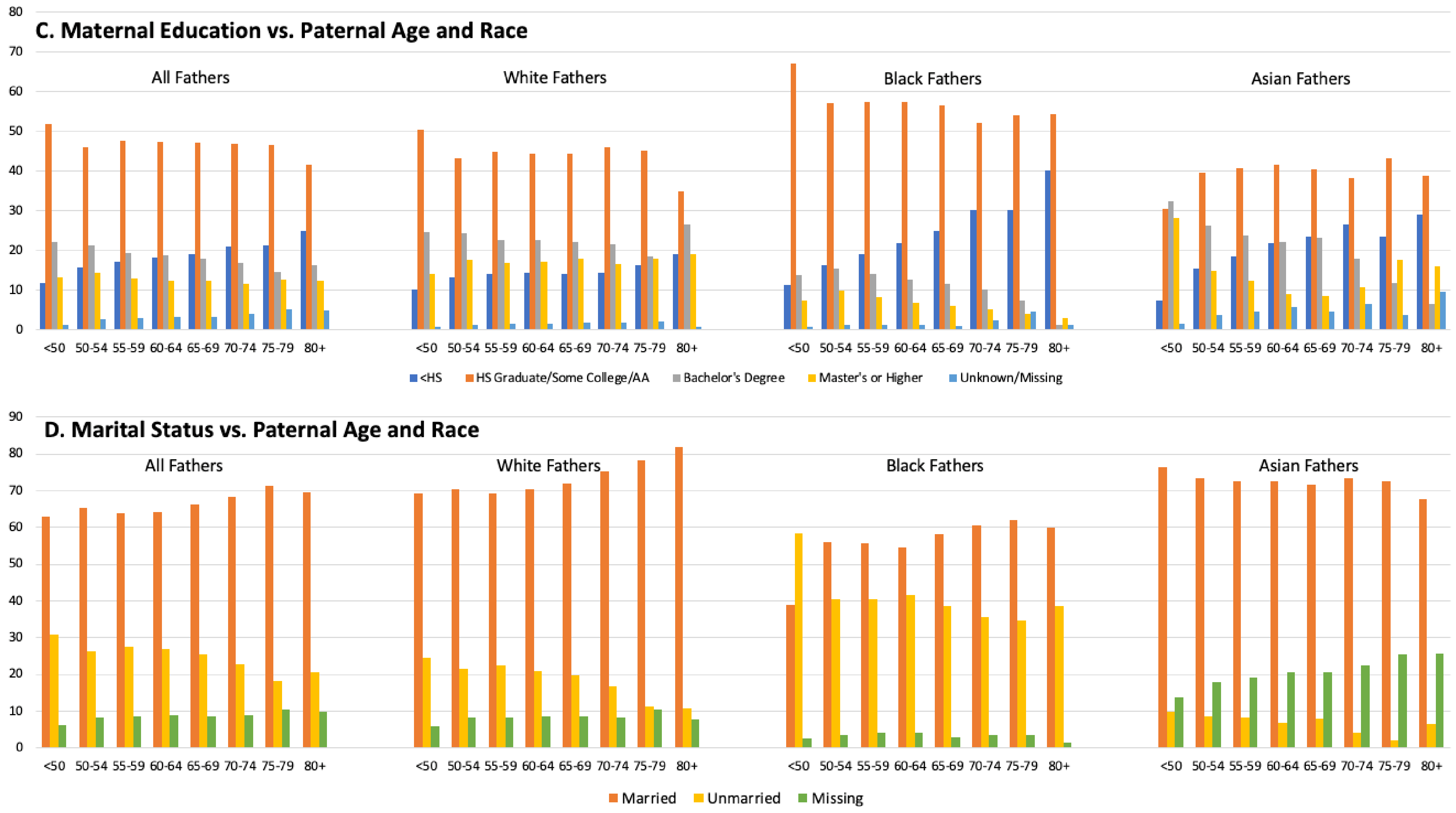

© 2024 Ha AS et al. *JAMA Network Open.*
