# Supplementary material for: Sociodemographic Trends and Perinatal Outcomes in Fathers 50 Years and Older
Source: JAMA Netw Open. 2024 Aug 1;7(8):e2425269. doi: 10.1001/jamanetworkopen.2024.25269 (PMC11294967; doi:10.1001/jamanetworkopen.2024.25269)
Supplement: Supplement 3. — Data Sharing Statement [file jamanetwopen-e2425269-s003.pdf]

Data Sharing Statement

Ha. Sociodemographic Trends and Perinatal Outcomes in Fathers 50 Years and Older. *JAMA Netw Open*. Published August 01, 2024. doi:10.1001/jamanetworkopen.2024.25269

Data
Data available: No

Additional Information
Explanation for why data not available: The data is publicly available and already deideintified in the NVSS (National Vital Statistics System)
